# Sickle cell disease patients in eastern province of Saudi Arabia suffer less severe acute chest syndrome than patients with African haplotypes

**DOI:** 10.4103/1817-1737.36550

**Published:** 2007

**Authors:** M. K. Alabdulaali

**Affiliations:** *Hereditary Blood Diseases Center, King Fahad Hospital, Hofuf, Saudi Arabia*

**Keywords:** Acute chest syndrome, sickle cell disease

## Abstract

**BACKGROUND AND OBJECTIVES:**

Genetic studies suggest that the sickle cell mutation has arisen on at least four separate occasions in Africa and as a fifth independent mutation in the Eastern Province of Saudi Arabia or India. The pathophysiology of sickle cell disease (SCD) is essentially similar in these different areas although the frequency and severity of complications may vary between areas. The aim of this study was to evaluate the prevalence and outcome of acute chest syndrome (ACS) in SCD patients from Eastern province of Saudi Arabia in comparison with patients with African haplotypes.

**MATERIALS AND METHODS:**

This was a retrospective study involving 317 SCD patients who were two years or older, admitted to King Fahad Hospital Hofuf between January-May 2003 for different etiologies. Twenty six patients presented with different causes of ACS; 11 patients presented with different pathologies other than ACS, but had past history of ACS; 280 patients presented with different pathologies and never presented with ACS. Clinical features, CBC, Hb-electrophoresis, G6PD activity, cultures, chest X-ray, arterial oxygen saturation, blood transfusion rates and outcome were studied. Univariate and multiple regression analysis were carried out to evaluate influence on ACS. Comparison between SCD patients with ACS from this study and from Eastern province of Saudi Arabia to patients with African haplotypes were carried out, using data reported in the literature.

**RESULTS:**

During the period of this study, 37 patients with new or previous episodes of ACS were studied (accounting for 11.67% of admitted SCD patients). Most of the patients with ACS had only one episode, but five patients (13.51%) had had episodes or more. One patient died giving an in-hospital mortality rate of 1/26 (3.85%). Comparison of recurrence of ACS and mortality between SCD patients in Eastern province of Saudi Arabia to that of patients with African haplotype showed that recurrence is significantly lower (*P*<0.025) in patients from Eastern province compared to patients with African haplotype, mortality also is lower but not statistically significant.

**CONCLUSION:**

Acute chest syndrome in SCD patients in Eastern province of Saudi Arabia is relatively uncommon, but causes significant morbidity and mortality. Its prevalence and recurrence is low if compared to that of patients with African haplotypes.

## Introduction

Sickle cell disease (SCD) is an autosomal inherited structural disorder of hemoglobin, associated with an amino acid substitution of valine for glutamic acid at the sixth residue of the ß-chain. This genetic alteration yields an unstable RBC with a shortened survival that under stress (e.g. deoxygenation) becomes sickle-shaped. The major consequence of this sickle shape is that RBCs become much less deformable; therefore, they obstruct the microcirculation. Tissue hypoxia, which promotes further sickling, results. The clinical manifestations of SCD are diverse and any organ system may be affected. These manifestations commonly are divided into vaso occlusive, hematologic and infectious crises. Evidence from structural studies of DNA suggest that the sickle cell mutation has arisen on at least four separate occasions in Africa and as a fifth independent mutation in the Eastern Province of Saudi Arabia or India.[1–3] Currently five haplotypes are known: Senegal haplotype, Benin haplotype, Bantu haplotype, Cameroon haplotype and Asian (Arab-India) haplotype from Eastern Province of Saudi Arabia and Central India.

The pathophysiology of SCD is essentially similar in these different areas although the frequency and severity of complications may vary between areas.[4,5]

Acute chest syndrome (ACS) is an acute pulmonary pathology that occurs in SCD patients. It is defined as a new infiltrate on chest radiograph in conjunction with other new symptom or sign: chest pain, cough, wheezing, tachypnea and/or fever (>38.5°C).[6] This condition is usually described in homozygous SCD, but it rarely develops in individuals with sickle-cell trait.[7] The term “acute chest syndrome” was first suggested for this complication by Charache *et al*.[8] in 1979, this term reflected the unique nature of acute pulmonary illness in patients with SCD and the difficulties in determining its pathogenesis. The frequency of this complication is variable, reaching up to 45% of individuals with SCD and recurring in up to 80% of those affected.[9–11] The incidence of ACS in patients with homozygous SCD is 12.8 episodes/100 patient years.[12] Incidence is inversely related to age, with children aged two through four years having the highest incidence (25.3 episodes/100 patient-years). Risk factors for the development of ACS include a high leukocyte count, low Hb-F concentration and a high hemoglobin level.[12]

Adult patients tend to have more severe course, death rate among them is higher than children, but ACS remains an important contributor to deaths at all ages, it accounts for 25% of deaths in SCD patients.[11,13–15]

In Hofuf area (Located in Al-Hassa region: Eastern province of Saudi Arabia) we have high prevalence of sickle cell gene; alpha, beta thalassaemia and Glucose 6 phosphate dehydrogenise (G6PD) deficiency, coexistence of these diseases in not uncommon. The frequency of sickle cell gene in Hofuf area ranges from 0.15-0.25. Homozygous SCD prevalence ranges from 1.0-1.5%.[16–18] It is believed that SCD patients in Hofuf and other areas in Eastern province of Saudi Arabia are having milder disease with less frequent complications and good outcome, due to the interaction between thalassaemia and SCD and for having Asian haplotype. The aim of the study was to evaluate the prevalence and outcome of ACS in SCD patients in Eastern province of Saudi Arabia and to compare it to patients with African haplotypes.

## Materials and Methods

A retrospective study of five months duration beginning January and ending May 2003 was carried out. The records of Saudi patients with SCD who were admitted to King Fahad Hospital, Hofuf for different aetiologies, during the period specified by the study were carefully studied. Three hundred and seventeen SCD patients were enrolled in the study. Their ages ranged between 2-50 years. Twenty six patients presented with different causes of ACS; 11 patients presented with different pathologies other than ACS, but had past history of ACS; 280 patients presented with different pathologies and never presented with ACS. Those admitted to this study fulfilled two criteria at presentation or on previous episodes: (a) new infiltrate on chest radiograph (X-ray) and (b) new respiratory symptom or sign mainly chest pain, cough, wheezing, tachypnea and/or fever (>38.5°C). Although it is reported that rare cases of ACS presented with normal chest X-ray,[19] but still the selected criteria is keeping with the most widely accepted definition of ACS.[6] Historical data, clinical features, CBC (using automated counter), Hb-electrophoresis (using sickling test and alkaline ± acid gel-electrophoresis methods), G6PD activity (using fluorescent screening test), Cultures (whenever needed), radiological studies such as; plain chest X-ray; abdominal ultrasound (measurement of splenic long axis length and evaluation of hepatobiliary tree), room air arterial blood gases (were done on admission whenever possible), blood transfusion rate/year, hospitalization rate, severity status (average number of acute admission / year for crisis, ACS, priapism, stroke, etc.)[20] and the outcome was studied.

Comparison between SCD patients with ACS from this study and from Eastern province of Saudi Arabia to patients with African haplotypes was carried out. Data obtained from references 21, 22, 23, 24 and 25.

The difference between ACS prevalence and outcome in the studied groups was determined by using *Chi^2^* analysis.

## Results

During the period of this study 317 SCD Saudi patients were studied. 37 patients with new or previous episodes of ACS were identified (account for 11.67% of admitted SCD patients). Most of the patients with ACS had only one episode, but five patients (13.51%) had two episodes or more, the number of admissions for each of these patients ranged from two to four (2 had 2 episodes, 2 had 3 episodes and 1 had 4 episodes). Thirty five (94.59%) are having homozygous SCD (Hb-SS), 2 (5.41%) are having sickle/B° (sickle/beta-thalassemia). Coexistence of G6PD deficiency was documented in 11 patients (29.73%).

Demographics, clinical features and hematological variables are shown in Table 1.

**Table 1:** Demographics, clinical features and hematological variables for sickle cell disease patients with acute chest syndrome

Variables	Group I (no.=26)	Group II (no.=11)
Age	Range: 2-50	Range: 5-19
Mean ± SD	14.2 ± 10.8	12.2 ± 4.7
12 years (%)	16/26 (61.5%)	7/11 (63.6%)
> 12 years (%)	10/26 (38.5%)	4/11 (36.4%)
Gender:		
Males:Females ratio	17:9 (1.9)	7:4 (1.8)
Hb (g/dl) Mean ± SD	8.5 ± 2.4	8.2 ± 1.1
MCV (fl) Mean ± SD	72.8 ± 9.7	72.8 ± 8.3
MCH (pg) Mean ± SD	24.8 ± 4.1	25.7 ± 4.4
WBC count (X109/L)		
Mean ± SD	14.2 ± 6.5	16.3 ± 8.2
Platelets count (X109/L)		
Mean ± SD	273.0 ± 164.1	304.0 ± 160.7
Hb-F (%) Mean ± SD	25.9 ± 10.5	26.1 ± 10.5
G6PD activity		
Deficiency (No.)	8/26 (30.77%)	3/11 (27.27%)
Splenic axis length (cm)		
Mean ± SD	12.3 ± 2.2	11.4 ± 2.1

Group I: Patients presented with different causes of ACS. Group II: 11 patients presented with different pathologies rather than ACS, but had past history of ACS.

**Figure 1:**
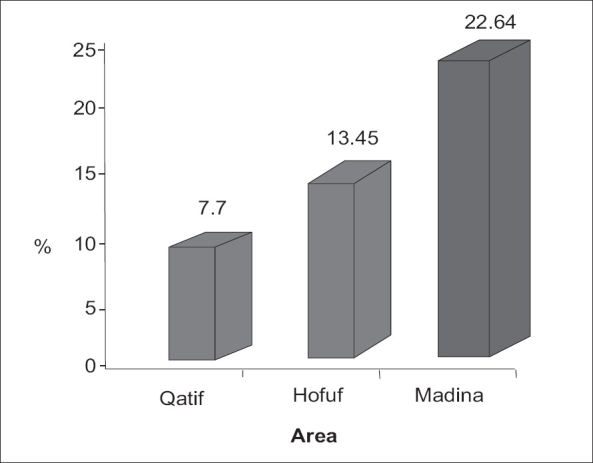
Prevalence of ACS in < 12 years old SCD patients in comparison between Hofuf, Qatif (located in Eastern province of Saudi Arabia) and Madina (located in Western area)

In this study the frequency of ACS among SCD patients in Hofuf was more frequent in younger patients <12 years than older patients 13.45% and 5.05% respectively. Castro *et al.* and Poncz *et al*., reported much higher frequency reaching up to 30-45% respectively.[9,12] Al-Dabbous reported 7.7% frequency In Qatif among patients younger than 12 years,[21] but in Madina (located in Western area of Saudi Arabia) the frequency of the same age group was reaching up to 22.64% as reported by Hawasawi *et al.*[22] [Figure 1]. If we look at the older patients in Hofuf it was reported by Al-Suliman *et al.*, that the frequency among patients older than 12 years was 1.38%[23] in comparison, it was reported that frequency in adult patients with African haplotypes reaches 9.63%.[24]

In this study, males were significantly affected more than the females in the previously mentioned from Hofuf and Qatif male predominance was reported with M:F ratio were 1.5 and 3 respectively.[21,23] On the other hand in patients with African haplotypes slight male predominance or even female predominance was reported.[24,25] Udezue *et al.*, reported some differences in the severity and features of SCD between males and females in Eastern area of Saudi Arabia.[26]

In our study the mean Hb-F level in SCD patients with ACS was 25.9% which higher than that reported in patients with African haplotypes 6%.[24]

In Qatif the average length of stay in hospital was 6.7 days with 11.68% of patients requiring ICU admission, patients with African haplotypes requiring an average stay of 10-11.4 days with 28% of patients requiring ICU admission.

**Figure 2:**
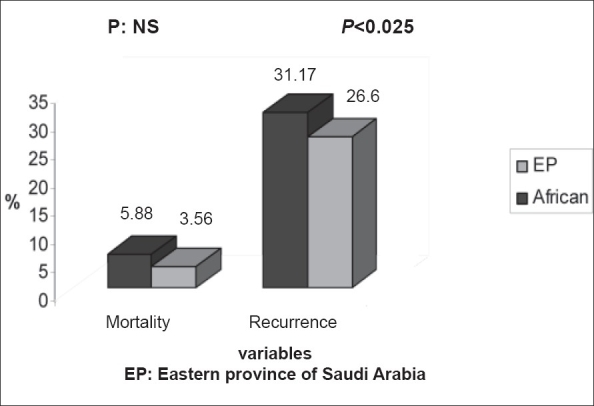
Rate of recurrence and mortality for ACS in comparison between SCD patients from Eastern province of Saudi Arabia versus patients with African haplotypes African: SCD patients with African haplotypes

Comparison of recurrence and mortality between SCD patients in Eastern province of Saudi Arabia to that of patients with African haplotype were carried out. I studied the summation of results in this study together with those done in Qatif and Hofuf previously[21,23] versus the results reported by Taylor, *et al.* and Bernard Maitre, *et al.*[24,25] Tables 2 and 3. I found that recurrence is significantly lower (*P*<0.025) in patients from Eastern province compared to patients with African haplotype, mortality also is lower but not statistically significant [Figure 2].

**Table 2:** Acute chest syndrome patients from Eastern province of Saudi Arabia versus patients with African haplotypes for recurrence and mortality

	Age group	Recurrence of ACS	References
Patients from Eastern province Adult of Saudi Arabia	Adult	29/109	23
Patients with African haplotypes	Adult	24/77	25

**Table 3:** Acute chest syndrome patients from Eastern province of Saudi Arabia versus patients with African haplotypes for mortality

	Mortality rate (episodes)	References
Patients from Eastern province of Saudi Arabia	13/(365)	21,23 and out study
Patients with African haplotypes	10/(170)	24 and 25

Genotype analysis was not included in this study. However; the clinical and laboratory features like splenomegaly and high Hb-F are highly suggestive of Asian haplotype predominance and/or coexistence of alpha-thalassaemia trait or hereditary persistent Hb-F.

## Discussion

SCD is one of the major health problems in Saudi Arabia, specially in Southern, Western and Eastern areas where the gene frequency of this disease is quite prevalent. Many studies were carried out in these areas to identify and compare the genetic variants and severity of SCD in between these areas. It is known that the Eastern areas are having different haplotypes than Southern and Western areas,[5,18,27,28] so we will try to discuss the prevalence, hospital course, recurrence and outcome of ACS in SCD patients in Eastern area -that include Hofuf and Qatif- where the Asian haplotype is more frequent and compare it with patients having African haplotypes including patients from Western area of Saudi Arabia where the African haplotypes are more frequent. ACS is less frequent in Eastern province than other areas where African haplotypes is frequent.

Length of stay in hospital and ICU admission is less in patients from Eastern province compared to patients with African haplotype.

Recurrence is significantly lower in patients from Eastern province compared to patients with African haplotype, mortality also is lower but not statistically significant.

These results support the theory that SCD patients in Eastern province has in general a milder disease -Avascular necrosis of femoral head and splenic complications are exceptions with less frequent ACS crisis, most likely due to high prevalence of Asian haplotype among SCD patients in this area,[5,18,21,27–30] coexisting alpha-thalassaemia,[31,32] persistence of high Hb-F levels,[33,34] haematology, social circumstances and geographical and climatic variation.[4,35]

## Conclusion

Acute chest syndrome in SCD patients in Hofuf area of Saudi Arabia is relatively uncommon, but causes significant morbidity and mortality. Its prevalence, recurrence and mortality are low if compared to that of patients with African haplotypes.

Future studies are needed to focus on diagnostic tools, course, management and outcome of this complication in SCD patients in Hofuf and other areas in Eastern province of Saudi Arabia with genotyping, using various biomarkers and including ECHO assessment.
